# First Field Test of the Novel Integration Mapping Tool for COVID-19 Vaccination Integration into National Immunization Programs and Primary Healthcare—A Case Study from Côte d’Ivoire

**DOI:** 10.3390/vaccines11121842

**Published:** 2023-12-12

**Authors:** Adidja Amani, Ekra Kouadio Daniel, Brou Gbotto, Kossia Yao, Aka Lépri Nicaise, Epa Kouakou, Kouadio Sie Kabran, Gervais Gahongano, Abdoul Gadiry Fadiga, Abdou Aziz Gbaya, Oniovo Efe-Aluta, Franck Mboussou, Imran Mirza, Benjamin Schreiber

**Affiliations:** 1 Faculty of Medicine and Biomedical Sciences, University of Yaoundé I, Yaounde 00237, Cameroon; 2 Ministry of Health Public Hygiene and Universal Health Coverage, Abidjan, Côte d’Ivoire; kdanielekra@yahoo.fr (E.K.D.); brougbotto_dr@yahoo.fr (B.G.); yaokossia@yahoo.fr (K.Y.); akanicaise@yahoo.fr (A.L.N.); 3 UNICEF Country Office Abidjan, Abidjan, Côte d’Ivoire; ekouacou@unicef.org (E.K.); agfadiga@unicef.org (A.G.F.); 4 WHO Country Office, Abidjan, Côte d’Ivoire; kouadios@who.int (K.S.K.); gahonganog@who.int (G.G.); 5 UNICEF West and Central Africa Regional Office, Dakar P.O. Box 29720, Senegal; agbaya@unicef.org; 6 WHO Inter-Country Support Team of West Africa, Ouagadougou, Burkina Faso; efeo@who.int; 7 WHO Inter-Country Support Team of Central Africa, Libreville, Gabon; mboussouf@who.int; 8 UNICEF HQ, New York, NY 10017, USA; imirza@unicef.org (I.M.); bschreiber@unicef.org (B.S.)

**Keywords:** COVID-19 vaccination, integration mapping tool, routine immunization, primary healthcare, field testing, Côte d’Ivoire

## Abstract

Introduction: With the containment of the COVID-19 pandemic in Côte d’Ivoire, efforts were made to seamlessly integrate COVID-19 vaccination into the national immunization program. A collaborative initiative involving UNICEF, WHO, GAVI, and partner organizations resulted in the creation of the COVID-19 Vaccine Integration Mapping Tool. This paper presents a case study documenting the field testing of the integration mapping tool and assessing the integration of COVID-19 vaccination within primary healthcare and routine immunization in Côte d’Ivoire. The study aims to describe the pilot process, gather feedback on tool usefulness and challenges, and establish integration priorities through roadmap development. Methods: Under the guidance of the Ministry of Health and Universal Coverage Cabinet, a workshop was conducted with participants from major health programs to field test the tool. Data analysis was performed using Excel, and the results were presented through tables, heat maps, and line graphs. Results: The first-of-its-kind field test of the integration mapping tool in Côte d’Ivoire showcased its potential to bring key partners together to discuss the current state of integration, improve transparency about resource allocation, and enhance data management for the incorporation of COVID-19 vaccination into existing immunization systems. The integration of COVID-19 vaccines in Côte d’Ivoire showed a moderate level of progress, with improvement needed in resource allocation, payment systems, targeting of highest-risk groups and vaccine administration. Support should be increased for target population identification, distribution points, quality of care mechanisms, and health personnel training. Health information systems and access to essential medicines were relatively satisfactory. Integration into existing programs, intersectoral collaboration, national health strategy, communication strategy, community participation, and data utilization require improvement. The post-workshop satisfaction survey gave the tool a score of 7 out of 10. Early lessons from Côte d’Ivoire provide guidance on enhancing integration, focusing on data-driven decision-making, collaboration, stakeholder engagement, and effective leadership. Conclusions: The field test of the integration mapping tool (IMT) in Côte d’Ivoire is groundbreaking as it exemplifies the transformative potential of innovative tools in immunization practices. Application of the IMT sets a precedent for seamless COVID-19 vaccination integration worldwide, emphasizing data-driven decision-making, collaboration, timing, and leadership. The success of the pilot exercise in Côte d’Ivoire was attributed to political commitment, well-facilitated workshops, assessments, and the fact that the team in the country had previously developed an initial integration plan.

## 1. Plain Language Summary

This paper communicates the outcome of a workshop held in April 2023 in Abidjan, Côte d’Ivoire, about the COVID-19 Vaccine Integration Mapping Tool (IMT), to be tested and evaluated in Côte d’Ivoire from 12 to 14 April 2023. As its name suggests, the tool aims to smooth the integration of COVID-19 vaccination into the national immunization program and primary healthcare system. The tool was developed through a collaboration between UNICEF, WHO, GAVI, and partner organizations, and it is part of the “Support package for integrating COVID-19 vaccination into immunization programmes and primary health care”. The researchers conducted a field test of the IMT to evaluate its effectiveness and gather feedback on its useability and functionality.

The results showed that the tool has the potential to improve coordination, resource allocation, and data management for the incorporation of COVID-19 vaccination into existing immunization systems and primary healthcare. The tool highlighted areas that needed improvement, such as resource allocation, payment systems, and vaccine administration.

The study also highlighted the importance of integrating COVID-19 vaccination into existing programs, promoting intersectoral collaboration, developing a national health strategy, implementing a communication strategy, encouraging community participation, and using data effectively. The post-workshop satisfaction survey gave the tool a score of 7 out of 10.

Overall, the field testing showed that the IMT Tool was effective in Côte d’Ivoire, demonstrating the potential of this innovative tool in transforming immunization practices. The findings provide valuable insights for improving integration efforts, and underline the importance of data-driven decision-making, collaboration, stakeholder engagement, and effective leadership.

This research contributes to the global understanding of integrating COVID-19 vaccination, and offers guidance for other countries making similar efforts. The lessons learned from Côte d’Ivoire’s experience can inform future advancements in integrated vaccination programs worldwide.

## 2. Introduction

The COVID-19 pandemic has presented challenges for African nations, including Côte d’Ivoire, which witnessed a toll with more than 88,000 reported cases and 834 fatalities as of 11 March 2023 [1]. In response, the country has implemented a range of strategies to combat the virus, notably an orchestrated vaccination campaign targeting individuals aged 12 and above, with particular emphasis on specific demographics such as 12–17-year-olds and expectant or nursing mothers [2]. Drawing on its expertise in the deployment of novel vaccines through the Expanded Immunization Program, Côte d’Ivoire has made progress in administering four distinct COVID-19 vaccines: Pfizer-BioNTech, AstraZeneca, Sinopharm, and Johnson & Johnson [2]. By March 2023, there had been progress in the national vaccination campaign targeting around 20 million individuals aged 12 and above. Of the total population of 30,215,522, 48% had received at least one dose of the vaccine, while 43% had completed their primary vaccination series. An allocation of 222,180 vaccine doses had also been dedicated to pregnant and lactating women [3].

Following a significant decline in COVID-19 cases, the National Security Council of Côte d’Ivoire decided to terminate the state of emergency on 12 April 2023 [4]. To ensure the sustained management of COVID-19, including the administration of booster shots to high-risk populations, the Security Council recommended that COVID-19 activities be included in routine healthcare by the end of June 2023. Early May 2023, the Director-General of the World Health Organization (WHO) declared that COVID-19 no longer constituted a public health emergency of international significance [5]. This shift made it necessary to integrate COVID-19 response measures into a comprehensive, long-term approach aimed at strengthening disease management. To facilitate this integration, UNICEF, WHO, GAVI, and partner organizations collaborated to develop the COVID-19 Vaccine Integration Mapping Tool (IMT) [6]. Aligned with the WHO health systems strengthening framework and part of the “Support package for integrating COVID-19 vaccination into immunization programmes and primary health care”, this tool encompasses essential components such as the health workforce, information systems, essential medicines, financing, leadership, governance, communication, and intersectoral collaboration. The WHO-UNICEF, in a four-step approach, outlined considerations for integrating COVID-19 vaccination [7], empowering countries to evaluate progress, identify areas for improvement, and formulate action plans.

Field testing of the tool was essential to ensure it was fit for purpose. In April 2023, the government of Côte d’Ivoire graciously extended an invitation to UNICEF and WHO for a collaborative mission. The Ministry of Health, taking the lead, convened all relevant departments in a workshop focused on integration. The objective of the mission was to field test the IMT and provide valuable assistance in facilitating the seamless integration of COVID-19 vaccines into routine immunization and primary healthcare systems. This paper documents a comprehensive case study to assess the effectiveness and feasibility of the IMT in facilitating the seamless integration of COVID-19 vaccination within Côte d’Ivoire’s existing immunization programs and primary healthcare system.

## 3. Objectives

This paper documents the field testing of the IMT in Côte d’Ivoire. More specifically, it aims to carry out the following:

1. Describe the piloting process and its outcomes.
2. Gather feedback on the tool’s usefulness and effectiveness, and potential challenges in its use.
3. Extract valuable lessons on useability and functionality from the early implementation of COVID-19 vaccine integration.

## 4. Methods

**Study Design**: The study is descriptive. A survey [8] was designed to collect participants’ ratings and feedback on the tool. The survey used a Likert scale ranging from 0 (lowest score) to 10 (highest score) to evaluate the tool’s usefulness.

**Setting**: The study was conducted in Côte d’Ivoire, a West African country covering a land area of 322,462 km^2^. It is bordered by the Atlantic Ocean to the south, Guinea and Liberia to the west, Mali and Burkina Faso to the north, and Ghana to the east. Côte d’Ivoire is divided into 31 regions, 111 departments, 509 sub-prefectures, and 201 municipalities [9]. The estimated population is 28,096,651, with 53.9% of inhabitants residing in urban areas [10]. The country has a poverty rate of 46.3% and a Gini index of 0.538, ranking 162 out of 189 countries [11]. The literacy rate in 2018 was approximately 65.2% for men and 44.8% for women [12].

**Health System**: The vaccination process in Côte d’Ivoire involves two main structures: the National Institute of Public Hygiene and Prevention (INHP) and the Expanded Program on Immunization (EPI). The INHP handles vaccinations for the general public, while the EPI focuses on children, pregnant women, and 9-year-old girls [13].

The EPI is implemented at all levels of the health system, including primary, secondary, and tertiary levels. Health centers, hospitals, and institutes provide immunization services, with district health systems overseeing

**Figure 1:**
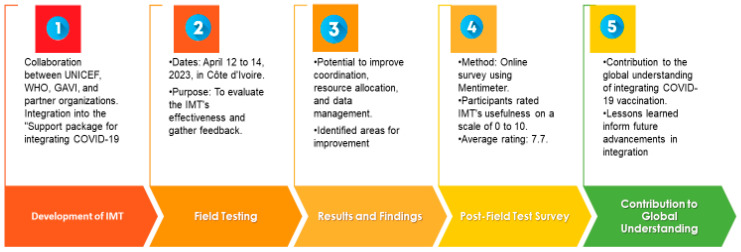
Flowchart provides a visual representation of the key phases and progression of the study, from its initiation to the dissemination of findings and lessons learned for future integration efforts.

Based on the evaluation of the process of implementation, the use of the IM tool, the result mapping, the survey, and the overall report of the workshop, it was possible to come up with a list of lessons learned from the whole exercise. The following aspects were detailed for enlightenment: participation, priority groups, collaboration, timing, technical support, innovation, and pillars for integration. All data analysis, with statistical evaluation, reporting and data visualization, was performed using Excel 5.0. The results were presented in tables, heat maps, and line graphs. The Ministry of Health supported the integration mapping tool pilot process as shown in Figure 1, including by providing official invitations. While no ethical clearance was required, participants consented to their experiences being documented and shared. Anonymized data were used solely for research and presented in aggregated form to protect privacy.

**Description of the Integration Mapping Tool**: The IMT is an Excel-based instrument comprising five main sheets that provide instructions, complementary materials, and assessment templates. It facilitates the integration of COVID-19 vaccination into national health systems by addressing dimensions such as leadership, collaboration, financing, service delivery, workforce, and information systems. The tool enables countries to assess their progress and generate visual summaries through heat maps, charts, and graphs, aiding decision-making and strategic planning. More information about the tool can be found on the TechNet-21 website [14]. Its implementation in Côte d’Ivoire marks a significant milestone and a pioneering field-testing site for integration of COVID-19 vaccination into existing immunization programs.

## 5. Field-Testing Process

The field-testing process for the IMT involved several steps conducted during a comprehensive workshop. The workshop encompassed multiple steps. The latter needed to be defined upfront before the commencement of the workshop by the expert group. Each step had to be clarified with links in between them, objectives, and expected outcomes.

## 6. Results

### 6.1. Participants

**Figure 2:**
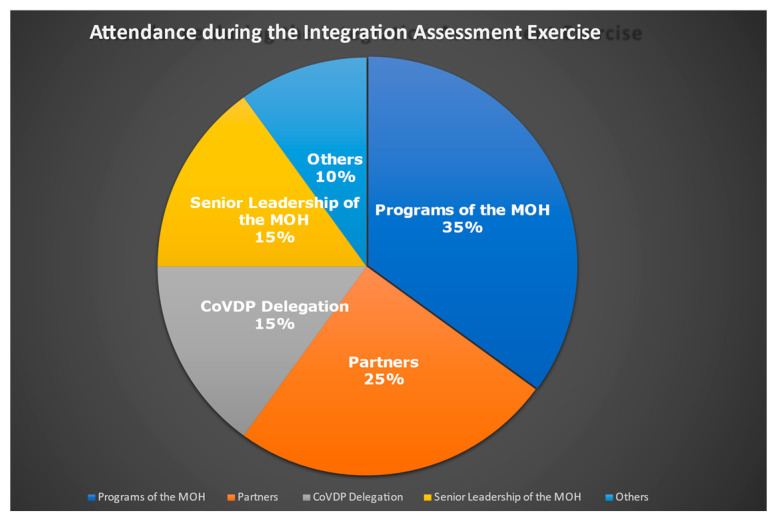
Mapping of participants in the integration assessment exercise.

The workshop with the meeting held at the Cabinet of the Ministry of Health witnessed the participation of 40 attendees, encompassing a diverse range of backgrounds and expertise. It brought together stakeholders from various levels, including representatives from central and operational tiers, healthcare programs, as well as technical and financial partners. The workshop saw six representatives from UNICEF Headquarters and Regional Office, WHO Africa Regional and Inter-Country Support Team of West Africa, and professionals from Boston Consulting Group (BCG). The attendees were further comprised of partners such as CDC, USAID, Agence de Médecine Préventive, Village Reach, and representatives from 14 priority programs of the Ministry of Health. Additionally, six participants from the Senior Leadership team at the Ministry of Health were present. The distribution of participants is illustrated in Figure 2.

### 6.2. The Process of Field Testing

**Figure 3:**
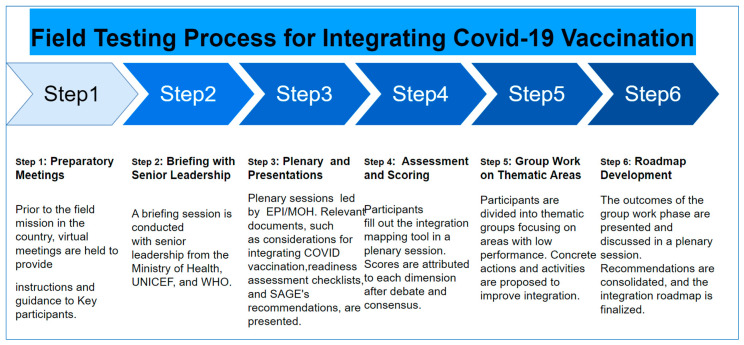
Streamlined approach to field testing COVID-19 Vaccination Integration Mapping Tool in Côte d’Ivoire.

The process developed by the team of experts has defined the following approach: first, there were preparatory meetings with high-level advocacy, followed by group-works, plenary presentations, and discussions. The final phase focused on designing an integration roadmap and providing debriefing to the authorities (see Figure 3).

The team took a six-step approach, starting with the preparatory meetings and ending with the validation of the integration roadmap.

**Step 1: Preparatory meetings**. To set the stage for a field test, the team organized virtual meetings weeks before traveling in country. At these meetings, the selected participants were given detailed guidance and instructions. The aim was to ensure a clear understanding of the objectives, methodologies, and expectations of the integration mapping tool.

**Step 2: Briefing with senior leadership**. In line with the Ministry of Health’s proactive approach to inclusive decision-making, a meeting was convened by the ministry itself, ensuring the participation of all relevant health programs. Representatives from key donor organizations and bilateral partners were also invited to join this session. The purpose of the briefing was to engage senior leadership from the Ministry of Health, UNICEF, and WHO in a constructive dialogue. The objective was to secure their commitment, gather their perspectives, and align the integration efforts with the broader health policies and strategies in place.

**Step 3: Plenary session and presentations**. A significant milestone in the field-testing process involved a plenary session, led by the Director of the Expanded Program on Immunization (EPI) and the official COVID-19 coordinator, who is also the technical advisor to the Minister of Health. During this session, directors of the different health programs and thus ‘high-level’ decision-makers were present. Key documents were presented, including considerations for integrating COVID-19 vaccination, readiness assessment checklists [14], and SAGE’s recommendations [15]. This comprehensive approach ensured that all participants, even those working on health programs which do not normally engage in vaccination, were well informed and equipped with the necessary knowledge.

**Step 4: Assessment and scoring**. Participants completed the IMT in a collaborative plenary session. Each dimension of the tool was evaluated based on the participants’ extensive experience and expertise. Debates and justifications ensued, ultimately leading to a consensus on the scores assigned to each dimension. This rigorous assessment process ensured the accuracy and reliability of the tool.

**Step 5: Group work on thematic areas**. To address areas with low performance, participants were divided into thematic groups. Each group focused on a specific area, such as community demand and engagement, leadership and governance, service delivery, or health financing. Through collective brainstorming, the groups proposed concrete actions and activities aimed at enhancing integration in these thematic areas.

**Step 6: Roadmap development**. Following the group work phase, the outcomes were presented and discussed in a plenary session. Recommendations from each thematic area were consolidated, taking into account the valuable insights and proposals generated during the group work. Through a meticulous process of analysis and refinement, the integration roadmap was finalized. This roadmap outlined the necessary steps and actions required to effectively integrate COVID-19 vaccination into routine immunization and primary healthcare.

To ensure the integration roadmaps were aligned with the overall integration goal, a synthesis meeting was scheduled after the mission. During this meeting, the roadmap was carefully validated and any necessary adjustments made. The team worked towards developing a comprehensive integration plan that encompassed all the dimensions addressed in the IMT. This plan was subject to further validation in a subsequent workshop, ensuring its robustness and effectiveness.

### 6.3. Readiness Mapping

**Figure 4:**
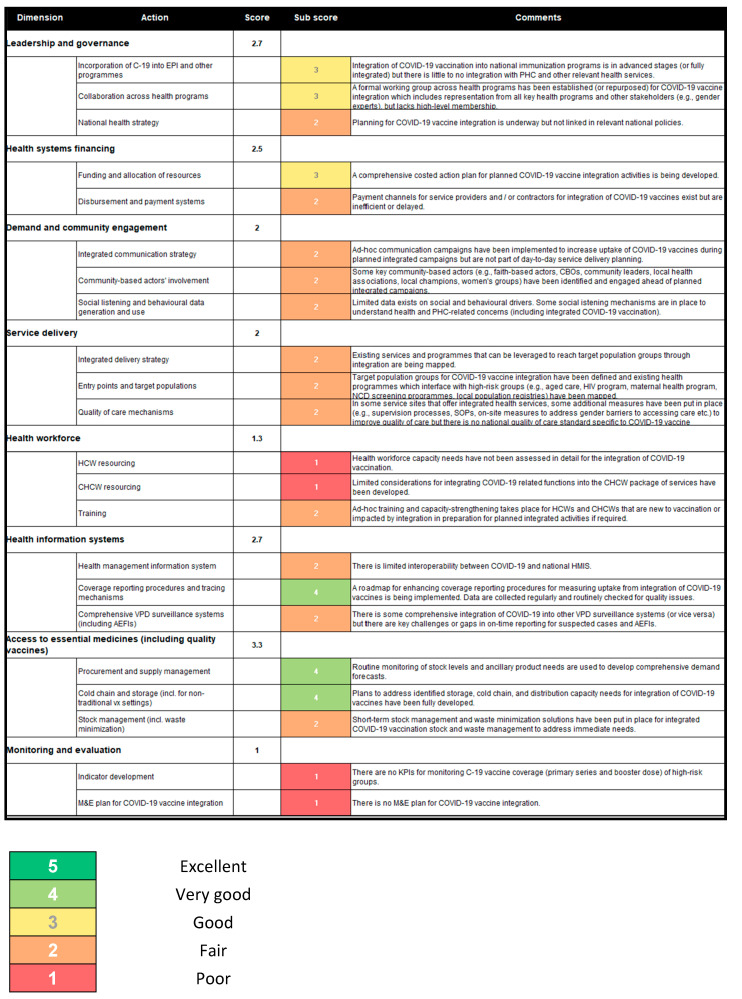
Heat map generated at the end of the scoring exercise.

The readiness of integrating the program of COVID-19 vaccination has been evaluated with the IM tool, as shown in Figure 4. Various dimensions and actions were assessed related to leadership and governance, program integration, intersectoral collaboration, healthcare financing, community engagement, service delivery, healthcare personnel, health information systems, access to essential medicines, and monitoring and evaluation. The results of the self-assessment tool for COVID-19 vaccine integration are presented in the form of scores for each dimension or action, ranging from 1 to 5. Based on these scores, we present a concise analysis to highlight the strengths, challenges, and opportunities for improvement in each area.

#### 6.3.1. Leadership and Governance

The integration of COVID-19 vaccines into routine immunization services (score: 3) has shown promising progress, particularly in target programs such as maternal health and school/university health. However, there is a need to extend this integration to primary healthcare services. Collaboration across sectors (score: 3) has been initiated through the establishment of an intersectoral task force. While key programs have been included, further efforts are required to involve additional programs such as those addressing HIV and malaria. The task force should be reoriented towards integration, and representation from higher-level officials should be expanded, for example through an expansion of the Interagency Coordinating Committee (ICC).

#### 6.3.2. Financing of Healthcare Systems

Efforts are underway in financing and resource allocation (score: 3), and a comprehensive action plan is being developed for the integration of COVID-19 vaccines. However, the current disbursement and payment systems (score: 2) lack efficiency. While the funding for integration is yet to be received, it is crucial to optimize existing systems to ensure effective use of resources.

#### 6.3.3. Community Engagement

Communication strategies (score: 2) have been implemented for integrated campaigns, including radio broadcasts and bus advertisements. However, a national strategy for integrated healthcare services is still lacking. Community stakeholders’ participation (score: 2) has been observed, particularly with key actors such as religious leaders, but efforts should be expanded to engage a broader range of community representatives. Social listening and behavioral data utilization (score: 2) have been undertaken through research in urban areas, but social identification mechanisms need further development if they are to provide more detailed information on primary healthcare concerns.

#### 6.3.4. Service Delivery

While an integrated implementation strategy (score: 2) has not been explicitly commented on, targeted population groups have been identified (score: 2). However, roles and responsibilities within these groups remain undefined. Mechanisms for improving quality of care (score: 2) have been implemented in selected integrated healthcare sites, but a national standard for quality care specific to COVID-19 vaccine integration is yet to be established.

#### 6.3.5. Healthcare Personnel

Assessment of healthcare workforce resources (score: 1) for COVID-19 vaccine integration is lacking in detail, although reports from specific services, such as the metabolic program, indicate a shortage of healthcare personnel. Special recruitment strategies are required to address this deficit. Availability of community health workers (score: 4) has been established through a georeferenced national reference list, but funding remains a persistent challenge. Training efforts (score: 2) require further exploration.

#### 6.3.6. Health Information Systems

Two parallel health information systems (score: 2) currently operate independently, and the COVID-19 system does not record vaccination characteristics on a daily basis. Notification procedures and tracing mechanisms (score: 4) exist but rely on manual transmission of data on request, indicating a need for automatic data exchange.

#### 6.3.7. Access to Essential Medicines

Effective management of procurement and supply (score: 4) is observed through regular stock monitoring at the central level. However, delays in order placement have been identified as an issue. Cold chain capacity and storage (score: 4) are established for COVID-19 vaccines, with ongoing assessments of existing storage capacity for integration purposes. Stock management policies (score: 2) exist but are not specifically tailored to COVID-19, such as ultra-cold chain requirements.

#### 6.3.8. Monitoring and Evaluation

The development of performance indicators (score: 1) for monitoring COVID-19 vaccine coverage and accurate denominators is still pending. A comprehensive monitoring and evaluation plan (score: 1) for COVID-19 vaccine integration is currently absent. Limited progress has been made in developing monitoring indicators.

The analysis of the readiness for integrating COVID-19 vaccination into the national immunization program reveals both strengths and areas for improvement. Progress has been made in certain dimensions, such as program integration and intersectoral collaboration, but several critical aspects, including healthcare personnel capacity, quality care standards, and monitoring and evaluation frameworks, require immediate attention.

## 7. Feedback and Insights from Participants on the Usefulness of the IMT

A survey was conducted among participants using the online platform Mentimeter [8], to assess overall opinions on the effectiveness of the IMT in supporting immunization integration efforts. The survey aimed to gather insights that could further improve the tool and enhance integration strategies. Participants were asked to rate the tool’s usefulness on a scale of 0 to 10, with 0 representing the lowest score and 10 the highest. The results of the survey revealed an average rating of 7.7 for the usefulness of the integration mapping tool. The survey ratings for this measure ranged from 5.5 to 10. This appreciation of the tool demonstrates its value in facilitating immunization integration efforts and supporting stakeholders in their decision-making processes. Participants reported that completing the integration mapping exercise using the tool gave them a comprehensive understanding of the existing integration landscape. By systematically mapping the integration dimensions and actions, the tool enabled stakeholders to identify both strengths and areas for improvement. This made it possible for them to take proactive measures to enhance integration efforts and address any gaps or challenges identified. The integration mapping tool also served as a baseline for measuring progress in immunization integration. It provided a structured framework that facilitated the evaluation of the effectiveness of interventions. Through the mapping process, stakeholders could assess the impact of various interventions and identify areas where further actions were needed.

## 8. Early Lessons Learned from Field Testing the Integration Mapping Tool for COVID-19 Vaccination

Some early lessons gained during the field-testing exercise include the importance of improving data collection processes, identifying priority areas, engaging multiple stakeholders, considering timing, providing necessary support, employing effective methods, and communicating with decision-makers. The following points summarize the early lessons learned.

## 9. Identifying Priority Groups for Effective Integration

The field testing in Côte d’Ivoire shed light on the challenges of identifying high-priority population groups and integrating pediatric populations and corresponding health services into COVID-19 vaccination efforts. The limited availability of data posed a significant obstacle in accurately assessing the progress of vaccination integration into routine and primary healthcare. To overcome these challenges, careful identification of high-priority groups and services is crucial for effective resource targeting and prioritization. The field-testing exercise served as a catalyst for prioritizing improvements in data collection and management processes, enabling better progress tracking and identification of areas for enhancement.

## 10. Harnessing Collaboration for Successful Integration

Collaboration emerged as a fundamental aspect of the integration process. Stakeholders from diverse sectors, including government agencies, healthcare providers, community leaders, and international partners, like WHO, UNICEF, USAID, CDC and Village Reach, actively participated in the development and implementation of integrated vaccination strategies. The inclusive approach fostered synergy, knowledge sharing, and resource pooling, resulting in a more cohesive and effective response to the vaccination needs of the population. The development of a roadmap for priority areas was made possible through systematic collaboration, leveraging the expertise and perspectives of stakeholders.

## 11. Maximizing Impact through Strategic Timing

The field-testing exercise conducted in April highlighted the challenges arising from timelines that conflict with pre-existing plans of primary healthcare programs. To optimize the integration of COVID-19 vaccination, it is crucial to schedule such exercises towards the end of the year when programs begin planning for the next cycle. This strategic timing enables seamless incorporation of COVID-19 vaccination integration into existing activities, ensuring maximum effectiveness and alignment with program objectives.

## 12. Enabling Success through Technical Support

The significance of support and facilitated discussions emerged as a key lesson during the integration process. Field missions with expert assistance proved invaluable in facilitating the adoption and use of the integration mapping tool. These missions contributed to the overall effectiveness of the integration efforts by providing necessary support, guidance, and expertise. Facilitating constructive discussions among participant groups also fostered a collaborative environment for idea exchange, consensus-building, and the identification of shared perspectives as well as divergent viewpoints. Recognizing the importance of technical assistance and promoting dialogue within stakeholder groups is crucial for empowering integration initiatives and maximizing their impact.

## 13. Unleashing Innovation through Group Workshops

The group workshops proved to be a pivotal factor in the integration process, facilitating productive exchanges, fostering diverse perspectives, and stimulating critical thinking. By asking participants to assess propositions from their peers, the workshops created an environment of peer learning and constructive criticism, allowing for a comprehensive evaluation of the integration process. The workshops encouraged participants to challenge ideas, leading to innovative solutions and a deeper understanding of the process.

## 14. Leadership, Understanding, and Ownership Are the Key Pillars of Successful Integration Efforts

Effective leadership at various levels of the healthcare system was instrumental in driving the successful integration of COVID-19 vaccination. Political commitment provided a strong foundation for the implementation of the IMT, ensuring the allocation of necessary resources and the prioritization of vaccination efforts. Furthermore, effective communication with decision-makers, such as government ministers, is vital. This requires clear and concise messaging tailored to the audience, accompanied by evidence-based and actionable recommendations. Strong political support and leadership create an enabling environment for integration efforts. Stakeholders involved in the integration must also have a comprehensive understanding of the complexities and nuances associated with merging different systems or programs. Furthermore, ownership of the integration process is essential, requiring sustained commitment from all stakeholders.

## 15. Discussion

### 15.1. Importance of the IMT Tool

The Integration Mapping Tool (IMT) played a critical role in assessing the current situation, shedding light on both strengths and weaknesses in the implementation of COVID-19 vaccination in Côte d’Ivoire. It provided a structured framework for evaluating the progress of integration into various health programs and emphasized key priorities. The IMT served as a catalyst for informed discussions and decision-making regarding integration priorities, ultimately leading to the development of a comprehensive integration roadmap. It is noteworthy that existing coordination mechanisms, such as the Interagency Coordination Committee (ICC), are set to be used for monitoring the integration roadmap’s implementation.

### 15.2. Value of the Workshop

To prepare for the implementation of the integration mapping tool, the country organized an integration planning workshop [16] involving multiple participants, which resulted in key recommendations. The integration planning workshop served as an invaluable platform for transparent information exchange among a diverse set of stakeholders. It facilitated open and constructive discussions that yielded key recommendations, contributing significantly to the improvement of COVID-19 vaccine integration efforts in Côte d’Ivoire. The workshop’s outcomes, guided by the insights from the IMT, marked a key step in enhancing access to COVID-19 vaccines for high-risk populations. It is worth noting that other countries have carried out integration exercises using the mapping tool, but through virtual meetings [17].

## 16. Need for Further Work on Implementation Plans

To ensure the effective reach of COVID-19 vaccines to high-risk populations through routine immunization and primary healthcare services, further work is imperative, particularly in the domains of financing and service delivery. It is essential to optimize resource allocation, payment systems, and vaccine administration. Supporting the identification of target populations, distribution points, quality of care mechanisms, and health personnel training is equally vital for successful integration. The IMT offers valuable insights into these areas, enabling targeted interventions and efficient resource allocation. While health information systems and access to essential medicines demonstrate relative strength, areas such as integration into existing programs, intersectoral collaboration, the national health strategy, communication strategy, community participation, and data utilization require improvements.

## 17. Analysis of Strengths and Weaknesses of Current Implementation Efforts

While certain dimensions of the integration process exhibit relative strength, others need enhancement. The IMT has revealed areas of strength in health information systems and access to essential medicines. In contrast, there are clear opportunities for improvement in aspects such as integration into existing programs, intersectoral collaboration, the national health strategy, communication strategy, community participation, and data utilization. Addressing these weaknesses is essential for a more robust and comprehensive integration of COVID-19 vaccination into the existing healthcare system.

The findings from this study underscore the potential of the IMT when used within a country-led process supported by high-level political commitment. The tool fosters collaboration across various primary healthcare programs, addressing the challenges related to integrating COVID-19 vaccination. It opens up new avenues for effective vaccination and improved healthcare delivery systems. The lessons learned from this case study provide valuable insights for accelerating progress towards vaccination targets and strengthening the integration process. It is worth noting that other countries have also used the IMT for integration exercises, albeit through virtual meetings.

The positive results of the survey underscore the utility and effectiveness of the IMT in supporting immunization integration efforts. It provides a comprehensive understanding of the integration landscape, identifies strengths and areas for improvement, and facilitates intervention evaluation. However, it is important to acknowledge that the integration exercise using the tool can be resource-intensive and time-consuming. Continuous communication and collaboration among stakeholders are essential for long-term sustainability. Furthermore, monitoring and evaluation, as well as regular updates to roadmaps, are crucial for adapting to new evidence and feedback.

This study boasts several strengths that enhance its credibility and relevance. The comprehensive approach, detailed documentation of the field-testing process, and utilization of a Likert scale survey for quantitative assessment contribute to its rigor. A wide range of data sources further enhances the study’s reliability and comprehensiveness. However, the study does have limitations, including its descriptive design, which restricts the establishment of causal relationships and raises concerns about generalizability due to sample size and participant representativeness. Overcoming these limitations requires further research, including controlled intervention studies, qualitative research methods, and comparative studies in diverse settings. Such research will provide stronger evidence and a more nuanced understanding of the IMT’s impact, ultimately advancing the field of COVID-19 vaccine integration and enhancing evidence-based decision-making and health system performance.

## 18. Conclusions

The implementation of the first field test of the Integration Mapping Tool (IMT) for COVID-19 Vaccination in Côte d’Ivoire is groundbreaking and holds immense promise. This research underscores the crucial importance of having strong country-led and managed processes to employ innovative tools like the IMT to engage different health programs in strategic discussions on improving access to COVID-19 vaccines for the highest-risk populations. The remarkable application of the IMT in Côte d’Ivoire paves the way for other countries to hold similar strategic dialogues to set up routine services within their immunization and primary health systems. By offering a standardized and systematic framework, the IMT facilitates these country-led dialogues and also ensures seamless integration into the health system. Key takeaways from the field test emphasize the significance of data-driven decision-making, collaboration, strategic timing, supportive environments, interactive workshops, and effective leadership. These invaluable insights and findings not only provide guidance for Côte d’Ivoire but also serve as a solid foundation for future advances in integrated vaccination programs, offering evidence-based support to nations embarking on similar integration endeavors.

## Data Availability

Data is contained within the article.
